# Attenuation of amiodarone induced lung fibrosis and phospholipidosis in hamsters, by treatment with the platelet activating factor receptor antagonist, WEB 2086

**DOI:** 10.1155/S0962935193000389

**Published:** 1993

**Authors:** S. N. Giri, D. M. Hyde, D. R. Haynam, M. Casias

**Affiliations:** 1 Departments of Veterinary Pharmacology and Toxicology University of California Davis CA 95616 USA; 2 Departments of Anatomy and Cell Biology School of Veterinary Medicine University of California Davis CA 95616 USA

## Abstract

Therapeutic use of amiodarone (AMD), a Class III antiarrhythmic drug is complicated by the development of lung fibrosis (LF) and phospholipidosis (PL). In the present study, the effectiveness of a PAF antagonist, WEB 2086, against AMD induced LF and PL has been tested in hamsters. The animals were randomly divided into four groups: (1) saline + H_2_O; (2) WEB + H_2_O; (3) saline + AMD; and (4) WEB + AMD. Saline or WEB (10 mg/kg i.p.) was given 2 days prior to intratracheal instillation of water or AMD (1.5 μmol/0.25 ml/100 g BW) and thereafter daily throughout the study. Twenty-eight days after intratracheal instillation, the animals were killed and the lungs processed for various assays. The amount of lung hydroxyproline, an index of LF, in saline + H_2_O, WEB + H_2_O, saline + AMD, and WEB + AMD groups were 959 ± 46, 1035 ± 51, 1605 ± 85 and 1374 ± 69 μg/lung, respectively. Total lung PL, an index of phospholipidosis, in the corresponding groups were 8.4 ± 0.4, 8.3 ± 0.3, 11.7 ± 0.3 and 9.9 μg/lung. Lung malondialdehyde, an index of lipid peroxidation and superoxide dismutase activity in saline + H_2_O WEB + H_2_O, saline + AMD, and WEB + AMD were 93.0 ± 4.3, 93.0 ± 2.7, 138.9 ± 6.0 and 109.0 ± 3.8 nmol/lung and 359.7 ± 13.9, 394.0 ± 22.8, 497.5 ± 19.7 and 425.5 ± 4.9 units/lung, respectively. Administration of AMD alone caused significant increases in all the above indexes of lung toxicity, and treatment with WEB 2086 minimized the AMD induced toxicity as reflected by significant decreases in these indexes. Histopathological studies revealed a marked reduction in the extent and severity of lung lesions in the WEB + AMD group compared with the saline + AMD group. Treatment with WEB 2086 also reduced the acute mortality from 35% in saline + AMD group to 22% in WEB + AMD group. It was concluded that PAF is involved in the AMD induced lung fibrosis and phospholipidosis and that the PAF receptor antagonist may, therefore, be potentially useful in reducing AMD induced lung toxicity.


Mediators of Inflammation 2, 279–285 (1993)

### Attenuation of amiodarone induced lung fibrosis and phospholipidosis in hamsters, by treatment with the platelet activating factor receptor antagonist, WEB 2086

S. N. Giri,[1,CA] D. M. Hyde,[2] D. R. Haynam[1] and M. Casias[1]

Departments of [1]Veterinary Pharmacology and Toxicology, and [2]Anatomy and Cell Biology School of Veterinary Medicine, University of California, Davis, CA 95616, USA

[CA] Corresponding Author

**THERAPEUTIC use of amiodarone (AMD), a Class III antiarrhythmic drug is complicated by the development of lung fibrosis (LF) and phospholipidosis (PL). In the present study, the effectiveness of a PAF antagonist, WEB 2086, against AMD induced LF and PL has been tested in hamsters. The animals were randomly divided into four groups: (1) saline + $H_2O$; (2) WEB + $H_2O$; (3) saline + AMD; and (4) WEB + AMD. Saline or WEB (10 mg/kg i.p.) was given 2 days prior to intratracheal instillation of water or AMD (1.5 $\mu$mol/0.25 ml/100 g BW) and thereafter daily throughout the study. Twenty-eight days after intratracheal instillation, the animals were killed and the lungs processed for various assays. The amount of lung hydroxyproline, an index of LF, in saline + $H_2O$, WEB + $H_2O$, saline + AMD, and WEB + AMD groups were 959 $\pm$ 46, 1035 $\pm$ 51, 1605 $\pm$ 85 and 1374 $\pm$ 69 $\mu$g/lung, respectively. Total lung PL, an index of phospholipidosis, in the corresponding groups were 8.4 $\pm$ 0.4, 8.3 $\pm$ 0.3, 11.7 $\pm$ 0.3 and 9.9 $\mu$g/lung. Lung malondialdehyde, an index of lipid peroxidation and superoxide dismutase activity in saline + $H_2O$, WEB + $H_2O$, saline + AMD, and WEB + AMD were 93.0 $\pm$ 4.3, 93.0 $\pm$ 2.7, 138.9 $\pm$ 6.0 and 109.0 $\pm$ 3.8 nmol/lung and 359.7 $\pm$ 13.9, 394.0 $\pm$ 22.8, 497.5 $\pm$ 19.7 and 425.5 $\pm$ 4.9 units/lung, respectively. Administration of AMD alone caused significant increases in all the above indexes of lung toxicity, and treatment with WEB 2086 minimized the AMD induced toxicity as reflected by significant decreases in these indexes. Histopathological studies revealed a marked reduction in the extent and severity of lung lesions in the WEB + AMD group compared with the saline + AMD group. Treatment with WEB 2086 also reduced the acute mortality from 35% in saline + AMD group to 22% in WEB + AMD group. It was concluded that PAF is involved in the AMD induced lung fibrosis and phospholipidosis and that the PAF receptor antagonist may, therefore, be potentially useful in reducing AMD induced lung toxicity.**

**Key words:** Amiodarone, Collagen, Lipid peroxidation, Lung fibrosis, Lung phospholipidosis, Platelet acting factor, Superoxide dismutase, WEB 2086

### Introduction

Amiodarone (AMD) was originally introduced as an anti-anginal drug,[1] but subsequently it was shown to possess an effective anti-arrhythmic property.[2] Currently, AMD is considered to be a Class III anti-arrhythmic drug and its use has been increasing in recent years for the treatment of resistant ventricular and supraventricular arrhythmia.[3,4] However, the clinical uses of this anti-arrhythmic drug are severely limited due to manifestations of serious pulmonary complications which range from subacute necrotizing pneumonitis to pulmonary fibrosis[5–8] as well as phospholipidosis.[9] Intratracheal (i.t.) instillation of AMD in hamsters as a single bolus induces pulmonary fibrosis which is initially characterized by interstitial pneumonitis that progresses to fibrosis.[10,11] Since the features of interstitial pulmonary fibrosis induced by i.t. instillation of AMD in hamsters resemble the idiopathic pulmonary fibrosis seen in humans, this model has been used for elucidating the underlying mechanisms of lung fibrosis and phospholipidosis and for evaluating the effectiveness of compounds as antifibrotic and antilipidotic agents.[12] Using the AMD hamster model of lung fibrosis and phospholipidosis, the effectiveness of a platelet activating factor (PAF) receptor antagonist, WEB 2086, as an antifibrotic and antilipidotic agent has been evaluated.

### Materials and Methods

*Animals and reagents:* Male Golden Syrian hamsters weighing 112–128 g were purchased from Sasco,

© 1993 Rapid Communications of Oxford Ltd

Inc. (Omaha, NE). The animals were housed four per plastic cage and had access to tap water and laboratory chow *ad libitum*. Bleomycin sulphate (Blenoxane[R]) was generously donated by Bristol Laboratories (Division of Bristol Myers Co., Syracuse, NY). L-[$^3$H](G)-hydroxyproline (specific activity 5 Ci/mmol) was obtained from ICN Radiochemicals (Irvine, CA) and hydroxyproline from Cal Biochem (San Diego, CA). Platelet activating factor (PAF) antagonist, WEB 2086, was a generous gift from the Boehringer Ingelheim Pharmaceutics, Inc. (Ridgefield, CT). All other reagents were of analytical grade and obtained from standard commercial sources.

*Treatment of animals:* Hamsters were housed in groups of four in plastic cages in facilities approved by the American Association for the Accreditation of Laboratory Animal care. They were acclimatized in a special room with constant temperature and filtered air for 1 week prior to the start of the experiment. A 12 h/12 h light/dark cycle was maintained and hamsters had access to water and Rodent Laboratory Chow 5001 (Purina Mills, Inc., St Louis, MO) *ad libitum*. The hamsters were randomly assorted into four experimental groups: (1) saline + water; (2) WEB + water; (3) saline + AMD; (4) WEB + AMD. WEB 2086 was dissolved in sterile isotonic saline and amiodarone hydrochloride in sterile distilled deionized water at 60°C. The AMD solution was stored at 4°C, protected from light. WEB 2086 (10 mg/kg) or an equivalent volume of saline was administered intraperitoneally once a day for 2 days prior to intratracheal instillation of AMD (1.5 $\mu$mol/0.25 ml/100 g BW) or an equivalent volume of water and thereafter daily throughout the study. The procedure of i.t. instillation was the same as described previously.[13] Briefly, hamsters were first anaesthetized with sodium pentobarbital (76–85 mg/kg i.p.) then AMD solution or an equivalent volume of $H_2O$ was instilled intratracheally by transoral route. All hamsters were sacrificed using an overdose of sodium pentobarbital (120–125 mg/kg) at 28 days following the i.t. instillation and their lungs were processed for morphological and biochemical studies.

*Processing of lungs for morphological studies:* After thoracotomy, the heart was ligated at its base to isolate the vasculature. The lungs were fixed by airway instillation with a cacodylate buffered glutaraldehyde–paraformaldehyde fixative (400 mOSm, pH 7.0) at 30 cm of $H_2O$ pressure. Lungs were fixed for at least 2 h, and blocks of tissue were cut from at least two sagittal slabs from the left, right cranial and right caudal lobes of each lung. Each slab was dehydrated in 95% ethanol and embedded in paraffin. Two sections from each lobe were cut and stained with haematoxylin and eosin. A lesion was defined as alveolitis consisting of thickened intra-alveolar septa resulting from oedematous swelling, fibrosis or the presence of inflammatory cells in either interstitium or airways.

*Processing of lungs for biochemical assay:* Lungs of each animal assigned to biochemical studies, were perfused *in situ* through the right side of the heart with 20 ml ice-cold isotonic saline. All lung lobes were quickly excised free of nonparenchymal tissue, washed in ice-cold saline, and then quickly frozen in liquid nitrogen before storing at −80°C. Subsequently, the frozen lungs were thawed and homogenized in 0.1 M KCl, 0.02 M Tris HCl buffer (pH 7.6) with a Polytron homogenizer (Brinkmann Instruments, Inc., Westbury, NY). After thoroughly mixing the homogenate, its total volume (10–11 ml) was recorded. Samples were divided into aliquots and stored at −70°C except the samples for lipid peroxidation and collagen assays, which were processed and assayed the same day the lungs were homogenized. The activity of superoxide dismutase (SOD) was determined on supernatant resulting from the centrifugation of the lung homogenate at 12 000 × ***g*** for 20 min at 4°C.

*Determination of hydroxyproline and prolyl hydroxylase activity:* For assay of lung hydroxyproline as a measure of collagen content, 1 ml of whole homogenate was precipitated with 0.25 ml of ice-cold 50% (w/v) trichloroacetic acid, centrifuged and the precipitate hydrolyzed in 2 ml of 6 N HCl for 18 h at 110°C. [$^3$H]-hydroxyproline (1 × $10^5$ dpm) was added to each sample as an internal standard to determine recovery and the hydroxyproline content was measured by the method of Woessner.[14] The hydroxyproline content for each sample was then corrected for its recovery which ranged from 75 to 85%. The method for propyl hydroxylase assay was the same as reported in a previous paper[15] and is based on the release of tritium from 3,4-[$^3$H]-proline labelled unhydroxylated procollagen as substrate prepared *in vitro* with 10-day-old embryonic chick tibiae. During the reaction, tritium is released in stoichiometric proportion to propyl hydroxylation as $^3H_2O$ which is counted and used as a measure of the prolyl hydroxylase activity. The activity was expressed as the total number of dpm released/lung/30 min.

*Determination of lipid peroxidation and SOD activity:* The lung malondialdehyde equivalent (MDAE) as an index of lipid peroxidation, was determined in the whole homogenate according to the method of Ohkakwa *et al.*[16] The SOD activity in the supernatant was measured from the rate at which the autoxidation of adrenaline to its adrenochrome was inhibited.[17] The rate of adrenochrome formation was 0.025 absorbance unit/min at 480 nm in a Varian carry 219 spectrophotometer (Beckman Instruments, Palo Alto, CA). Under these defined conditions, the amount of tissue required to inhibit the rate of adrenochrome formation by 50% (i.e. 0.0125 absorbance unit/min) was defined to contain 1 unit of SOD activity.

*Determination of lung phospholipid:* The total phospholipid from the whole lung homogenate was extracted in a mixture of chloroform and methanol using the method of Bligh and Dyer[18] with slight modification to maximize the phospholipid recovery. Briefly, 0.5 ml of the homogenate was mixed with 2 ml of chloroform, 2 ml methanol and 1.3 ml of double distilled water and homogenized with a Polytron. After separating the two phases by centrifugation, the upper alcoholic phase was gently aspirated and discarded followed by addition of another 1 ml of chloroform. Samples were vortexed and the two phases were separated by centrifugation. After removing the upper phase, the chloroform layer containing the phospholipid was evaporated to dryness under nitrogen. Total lipid phosphorous was quantitated by the method of Ames and Dubin.[19] Phospholipid values are reported as micromoles per total lung.

*Statistical analysis of data:* All data are expressed on the basis of total lung. Expression of the data on a per lung basis avoids the artifactual lowering of the values in AMD treated animals due to the presence of proteins of extrapulmonary origin.[20] Values are reported as the mean ±S.E.M. and the data were analysed by a one-way analysis of variance and Fisher's least significant difference multiple comparison text (Number Cruncher Statistical Analysis System Version 5.1). A value of $p \leq 0.05$ was considered significant.

### Results

*Body weight:* The effect of daily administration of WEB 2086 on body weight of hamsters with and without i.t. instillation of amiodarone is shown in Fig. 1. Although all amiodarone treated animals initially lost weight thereafter, they gradually gained weight and caught up with the hamsters in either saline or WEB control groups at 2 weeks after the i.t. instillation. In fact there were no significant differences in body weight among the four groups of hamsters at the time the experiment was terminated.

*Mortality:* Intratracheal instillation of AMD caused a 35% mortality (seven out of 20 died) in the saline + AMD group within 48 h post-treatment as opposed to 22% mortality (four out of 18 died) in the WEB + AMD group over the same period of time. There was no mortality in saline + water (0/12) and WEB + water (0/12) groups at any time of the study.

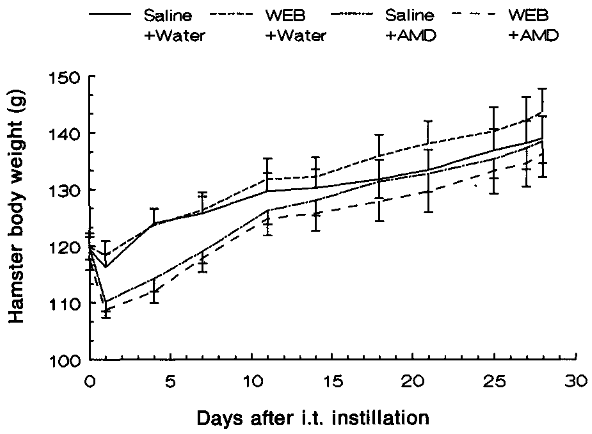

FIG. 1. Effects of intratracheal instillation of water or amiodarone (AMD) with or without treatment of WEB 2086 on body weight of hamsters. WEB 2086 (10 mg/kg) or an equivalent volume of saline was injected intraperitoneally once a day for 2 days prior to intratracheal instillation of AMD (1.5 μmol/0.25 ml/100 g BW) or an equivalent volume of sterile distilled water and thereafter daily throughout the study. Hamsters were weighed twice a week and on the 28th day, the experiment was terminated. Each point represents the mean ±S.E.M. of 12–14 animals in each group.

*Lung hydroxyproline content and prolyl hydroxylase activity:* The effects of daily treatment with WEB 2086 on AMD induced increases in lung hydroxyproline and prolyl hydroxylase activity are shown in Figs 3 and 4, respectively. Intratracheal instillation of AMD significantly increased the lung hydroxyproline content to 167% of saline control. Treatment with WEB 2086 caused a significant attenuation in the AMD induced increase in lung hydroxyproline content in the WEB + AMD group, although the hydroxyproline level in the latter group was still significantly higher than that of saline + $H_2O$ or WEB + $H_2O$ group (Fig. 2). Similarly, i.t. instillation of AMD alone increased the lung prolyl hydroxylase activity significantly to 218% of the

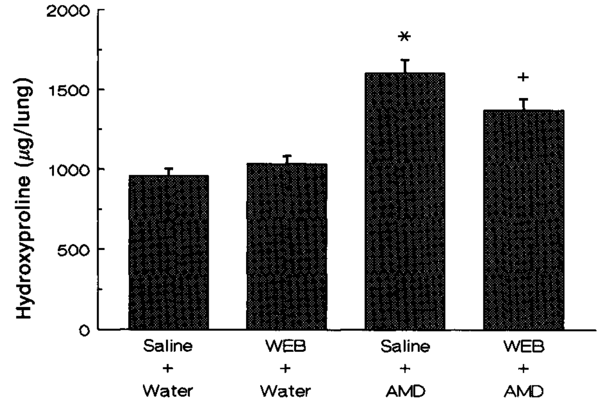

FIG. 2. Effects of WEB 2086 on amiodarone (AMD) induced increases in hydroxyproline content of hamster lungs. See the legend to Fig. 1 for treatment details. Each value represents the mean ±S.E.M. of eight animals in each group. * Significantly higher ($p \leq 0.05$) than all other groups. † Significantly higher ($p \leq 0.05$) than saline + water and WEB + water groups.

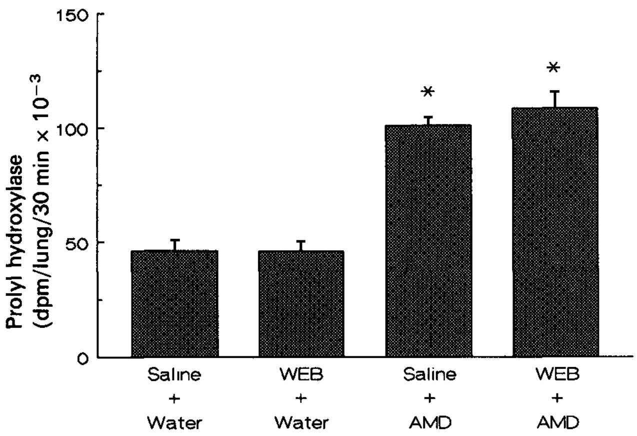

FIG. 3. Effects of WEB 2086 on amiodarone (AMD) induced increases in prolyl hydroxylase activity of hamster lungs. See the legend to Fig. 1 for treatment details. Each value represents the mean ±S.E.M. of eight hamsters in each group. * Significantly higher ($p \leq 0.05$) than saline + water and WEB + water groups.

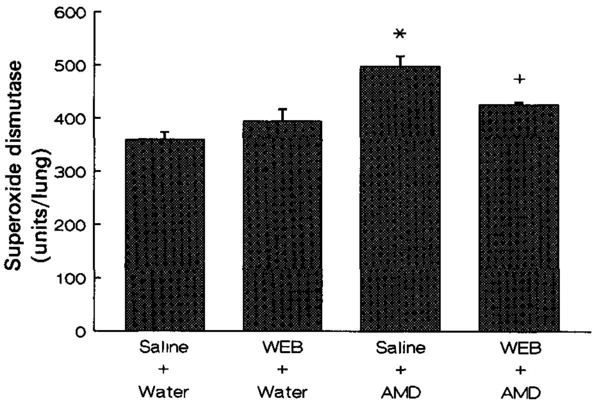

FIG. 5. Effects of WEB 2086 on amiodarone (AMD) induced increases in superoxide dismutase activity of hamster lung. See the legend to Fig. 1 for treatment details. Each value represents the mean ±S.E.M. of eight hamsters in each group. * Significantly higher ($p \leq 0.05$) than all other groups. † Significantly higher ($p \leq 0.05$) than saline + water group but not WEB + water group.

saline + $H_2O$ group. However, daily treatment with WEB 2086 had no effect on AMD induced increase in the lung prolyl hydroxylase activity in the WEB + AMD group. In fact the activity in the latter group of animals was significantly increased to 235% of the saline control. There was no significant difference in the lung prolyl hydroxylase activity between the saline + AMD and WEB + AMD groups (Fig. 3).

*Lung MDAE and SOD activity:* Intratracheal instillation of AMD significantly increased the lung MDAE content to 149% of saline + $H_2O$ control at 28 days post-treatment (Fig. 4). Administration of WEB 2086 once a day over the same period caused a significant reduction in the AMD induced increase in the lung MDAE content in the WEB + AMD group, although the MDAE level in this group was still significantly higher than that of saline + $H_2O$ or WEB + $H_2O$ control group. The lung SOD activity in the AMD treated group was also significantly increased to 138% of saline + $H_2O$ control and treatment with WEB 2086 attenuated significantly the AMD induced increase in the lung SOD activity in the WEB + AMD group, although the activity in this group remained significantly higher than that of saline + $H_2O$ but it did not differ significantly from that of the WEB + $H_2O$ group (Fig. 5).

*Lung total phospholipid content:* The effect of i.t. instillation of AMD in hamsters with and without WEB 2086 treatment is summarized in Fig. 6. There was a significant increase in the total phospholipid content of the lungs to 138% of the saline + $H_2O$ control and treatment with WEB 2086 reduced the AMD induced increase in the lung phospholipid level significantly in the WEB + AMD group, although the level in this group was still significantly higher than that of saline + $H_2O$ or WEB + $H_2O$ groups.

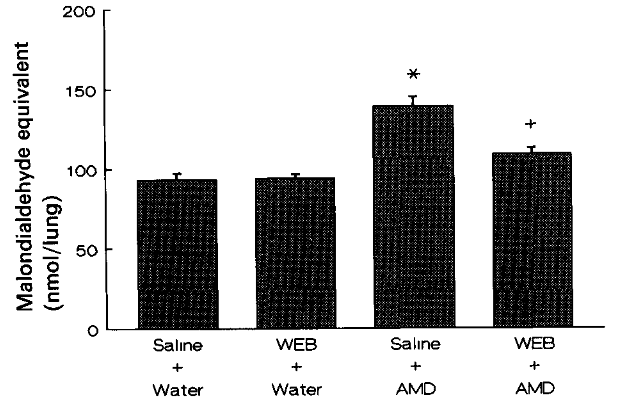

FIG. 4. Effects of WEB 2086 on amiodarone (AMD) induced increases in malondialdehyde equivalent level in hamster lung. See the legend to Fig. 1 for treatment details. Each value represents the mean ±S.E.M. of eight hamsters in each group. * Significantly higher ($p \leq 0.05$) than all other groups. † Significantly higher ($p \leq 0.05$) than saline + water and WEB + water groups.

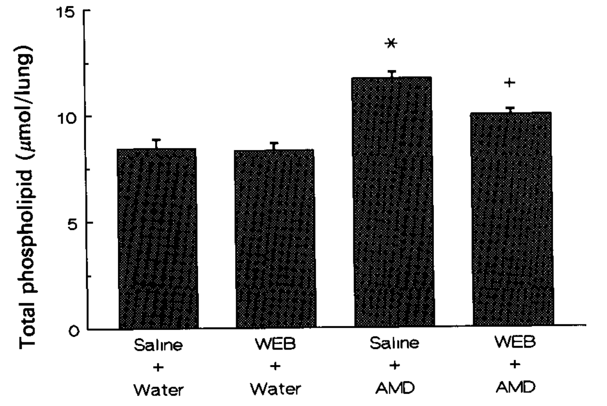

FIG. 6. Effects of WEB 2086 on amiodarone (AMD) induced increases in total phospholipid content of hamster lung. See the legend to Fig. 1 for treatment details. Each value represents the mean ±S.E.M. of eight hamsters in each group. * Significantly higher ($p \leq 0.05$) than all other groups. † Significantly higher ($p \leq 0.05$) than saline + water and WEB + AMD groups.

*Histopathology:* The histopathological study was performed in four to six hamsters per group. Histopathological evaluation of saline + $H_2O$ and WEB + $H_2O$ groups revealed normal pulmonary parenchymal tissue which was characterized by a lack of any notable aggregation of inflammatory cells in alveolar spaces or the interstitium, and thin interalveolar septa (Fig. 7A,B). In contrast, lungs from hamsters in the saline + AMD group had lesions that ranged from multifocal proximal acinar interalveolar septal thickening and aggregations of airway inflammatory cells to multifocal fibrotic lesions that obliterated airspaces and showed abundant fibroblasts and collagen (Fig. 7C). On average, about 10% of each lobe had pulmonary lesions in this group. The pulmonary lesions in the hamsters of WEB + AMD group were limited to multifocal proximal acinar, interalveolar septal thickening and aggregation of airway inflammatory cells and some peripheral interalveolar septal thickening with more mononuclear inflammatory cells and less fibroblasts and collagen in the interstitium (Fig. 7D). There was a marked reduction in the extent and severity of the lesion in the WEB + AMD group compared with the saline + AMD group.

### Discussion

Consistent with the authors' previous study as well as with the studies of other investigators, i.t. instillation of AMD caused significant increases in lung collagen and phospholipid content.[10-12] In the present study, earlier findings that AMD treated hamsters had twice as much prolyl hydroxylase activity per lung as the hamsters in control groups were also confirmed.[12] There are several proposed underlying mechanisms for the pathogenesis of AMD induced lung injury,[21-23] including oxidation via generation of reactive oxygen species (ROS).[24] The potential biochemical mechanisms responsible for the oxidant effect of AMD is not known. Li and Chignell[25] found that UV irradiation of AMD and its desethyl metabolite produced a carbon-centred radical capable of abstracting a hydrogen atom from linoleic acid and forming the corresponding pentadienyl radical. Reaction of the pentadienyl radical with oxygen *in vivo* would yield a peroxyl radical responsible for propagation of a chain of lipid peroxidation.

It is conceivable that PAF may be one of the products resulting from the peroxyl radical induced oxidation of the phospholipid membrane of cells.

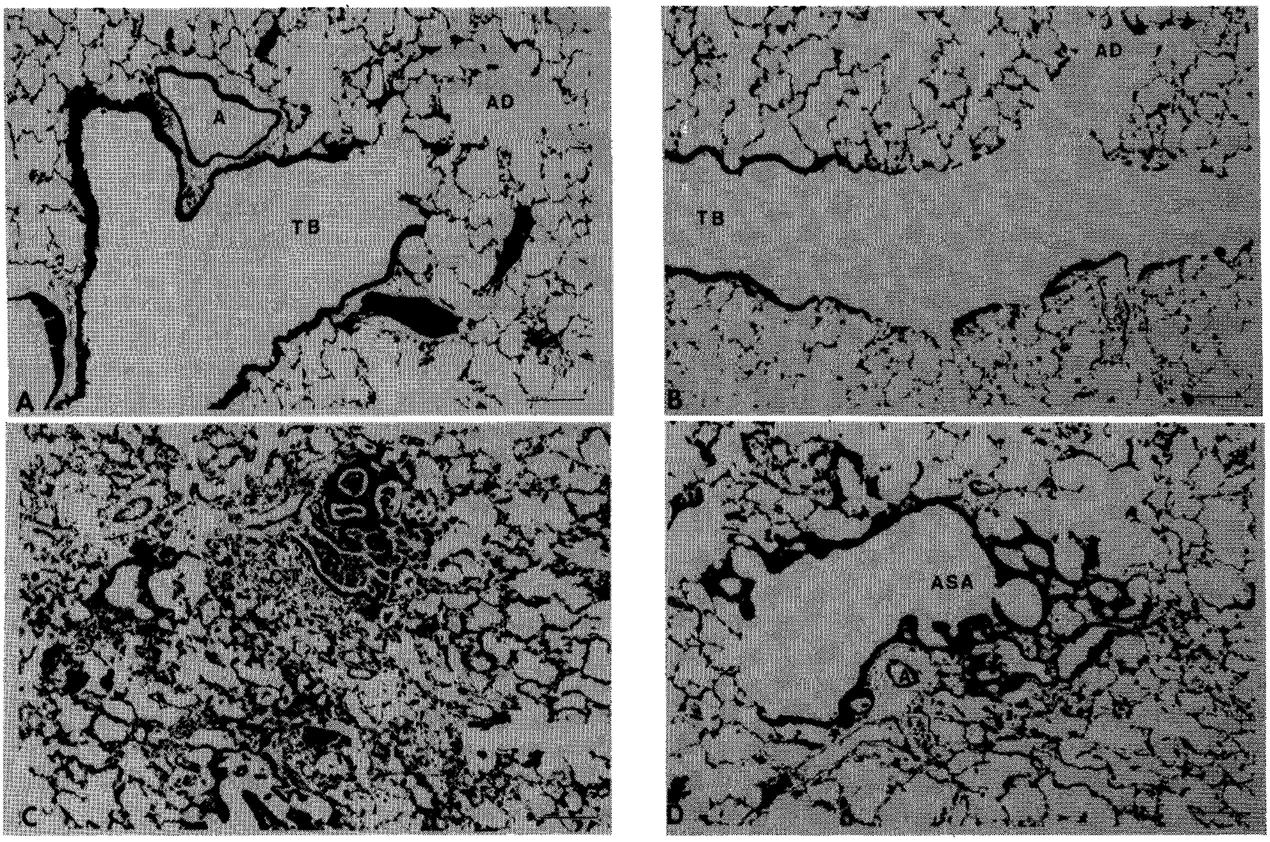

FIG. 7 Effects of WEB 2086 on amiodarone (AMD) induced parenchymal lesions of hamster lungs. See the legend to Fig. 1 for treatment details. (A) A representative micrograph of lungs from hamsters in the saline + water group shows normal terminal broncheoles, alveolar ducts and thin interalveolar septa. (B) A representative micrograph of lungs from hamsters in the WEB + water group shows normal terminal bronchioles, alveolar ducts and thin interalveolar septa. (C) A representative micrograph of lungs from hamsters in the saline + AMD group shows abnormal small airways with aggregations of airway inflammatory cells (arrowheads). Note the adjacent areas of thickened septa and organized connective tissue. (D) A representative micrograph of lungs from hamsters in the WEB + AMD group shows an abnormal small airway and a few thickened septa. Note the reduced severity of inflammation and fibrosis in this treatment group. A = arteriole, TB = terminal bronchiole, AD = alveolar duct, ASA = abnormal small airways, CT = organized connective tissue, bar = 100 $\mu m$.

This hypothesis is based on evidence available in literature that ROS such as $H_2O_2$ stimulates the synthesis of PAF in primary cultures of bovine pulmonary artery endothelium and human umbilical vein endothelium.[26] Compared with other lipid mediators of inflammation, such as those generated via the cyclooxygenase or lipoxygenase pathway, PAF has a wider range of inflammatory effects on many cell types including activation of neutrophils, eosinophils, lymphocytes and macrophages. The stimulation of neutrophils by PAF results in the release of lysosomal enzymes and superoxide anions and the generation of $LTB_4$.[27–29] PAF also increases the production of superoxide anion by human alveolar macrophages in a dose dependent manner.[30]

Our data support the notion that PAF is involved in the AMD induced lung toxicity via generation of superoxide radicals. Involvement of this radical is indirectly suggested by the increase in lung superoxide dismutase (SOD) activity that was found after AMD treatment. Increase in the SOD activity is presumably a compensatory defence mechanism to protect tissue against the deleterious effects of superoxide radicals.[31] However, the increase in the activity of this enzyme as a consequence of enhanced cellularity in the lung due to inflammatory and reparative processes cannot be ruled out. Increase in the lung MDAE content which is conventionally used as an index of lipid peroxidation, that was found after AMD also suggests the role of superoxide radical in AMD induced lung toxicity. Furthermore, it has been demonstrated that superoxide anions stimulate collagen synthesis by modulating differential collagen gene expression of fibroblasts in culture.[32] This finding may help explain the AMD induced increase in the lung collagen content as found in the present study.

The biochemical findings that WEB offers protection against AMD induced lung toxicity are in corroboration with the histological findings since hamsters in the WEB + AMD group showed a marked reduction in the extent and severity of lung inflammation and fibrosis as compared with hamsters in saline + AMD group. It appears that generation of superoxide anions resulting from activation of lung neutrophils and macrophages by PAF could be one of the possible factors in the pathogenesis of AMD induced lung fibrosis and phospholipidosis. This was substantiated by the biochemical and histological findings of the present study that PAF receptor antagonist (WEB 2086) attenuated most of the indices of lung toxicity caused by i.t. instillation of AMD.

It is surprising that WEB 2086 caused significant reductions in AMD induced increases in the lung collagen and phospholipid content but it had no effect on increased lung prolyl hydroxylase activity. The prolyl hydroxylase catalyses hydroxylation of proline residues on nascent procollagen polypeptides before helix formation and secretion.[33] Although this is not a rate-limiting step in collagen synthesis,[34] increased activity of this enzyme frequently predates collagen accumulation in experimental models of fibrosis.[15,35] However, it should be noted that tranilast, which is used for treatment of bronchial asthma and allergic rhinitis, was found to specifically suppress the collagen synthesis of fibroblast from keloid and hypertrophic scar tissue but had no effect on prolyl hydroxylase activity except at an extremely high concentration.[36]

There are several lines of evidence which suggest that increased production of PAF may be responsible for a variety of inflammatory conditions. For instance, increased production of PAF as reflected by its elevated blood level was implicated in the impaired haemodynamics of cirrhotic patients.[37] Recently, Zhou *et al.*[38] demonstrated that increased production of PAF from Kupffer cells may be an important mediator for the hepatic inflammation resulting from a short-term ligation of bile duct. An increased production of PAF has been implicated in the pathophysiology of a variety of pulmonary disorders including airway microvascular leakage, mucous production, bronchial asthma, adult respiratory distress syndrome and pulmonary hypertension.[39] It has been suggested that PAF is also involved in the bronchopulmonary dysplasia resulting from chronic lung injury which eventuates in airway obstruction, pulmonary oedema, pulmonary hypertension and interstitial fibrosis.[40]

Interaction of PAF with its specific receptor promotes multiple biochemical pathways, each of which contributes to the process of cellular activation.[41] These pathways include activation of a GTPase that inhibits adenylate cyclases; stimulation of $Ca^{2+}$ stores and activation of phospholipases that yield diacygIycerol, inositol triphosphate and free arachidonic acid which can be acted upon through the cyclooxygenase and lipoxygenase pathways to give eicosanoids.[42] It is interesting and consistent with the above PAF receptor interaction hypothesis that the authors previously reported an inhibition of adenylate cyclase,[43] increased intracellular level of calcium,[44] increased lung phospholipase $A_2$ activity[45] and increased plasma[46] and lung[47] levels of some eicosanoid in a bleomycin hamster model of pulmonary fibrosis.

The findings reported in this study that the PAF receptor antagonist (WEB 2086) not only offered protection against the acute toxicity but it also ameliorated lung fibrosis and phospholipidosis at 28 days after i.t. instillation of AMD, suggest that PAF is involved in the pathogenesis of fibrotic and phospholipidotic processes. The results are consistent with the results of Rodriguez-Barbero *et al.*[48] who found that treatment with the PAF receptor antagonist, BN 52021, markedly attenuated the gentamicin induced nephrotoxicity in rats. However, the present findings should be interpreted cautiously with respect to the effectiveness of WEB 2086 in attenuating the AMD induced lung fibrosis and phospholipidosis in humans in therapeutic situations for two reasons. First, i.t. instillation of AMD in hamsters does not simulate oral administration of this drug in humans and second, the fibrosis that results in hamsters may not be comparable to what occurs in humans in clinical situations. Nevertheless, use of WEB 2086 in the AMD hamster model of lung injury has opened a potentially novel approach in the management of lung fibrosis and phospholipidosis.

### References

1. Singh BN. Amiodarone: historical development and pharmacological profile. *Am Heart J* 1983; **106**: 788–797.
2. Rosenbaum MB, Chiale PA, Halpern MS, *et al.* Clinical efficacy of amiodarone as an anti-arrhythmic agent. *Am J Cardiol* 1976; **38**: 934–944.
3. Leak D, Eydt JN. Control of refractory cardiac arrhythmias with amiodarone. *Arch Intern Med* 1979; **139**: 425–428.
4. Heger JJ, Prystowsky EN, Zipes DP. Clinical efficacy of amiodarone in treatment of recurrent ventricular tachycardia and ventricular fibrillation. *Am Heart J* 1983; **106**: 887–894.
5. Martin WJII, Standing JE. Amiodarone pulmonary toxicity: biochemical evidence for a cellular phospholipidosis in the bronchoalveolar lavage of human subjects. *J Pharmacol Exp Therap* 1988; **244**: 774–779.
6. Marchlinski FE, Gansler TS, Waxman HL, Josephson M. Amiodarone pulmonary toxicity. *Ann Intern Med* 1982; **97**: 839–845.
7. Sobol SM, Rakita LL. Pneumonitis and pulmonary fibrosis associated with amiodarone treatment: A possible complication of a new anti-arrhythmic drug. *Circulation* 1982; **65**: 819–824.
8. Primeau R, Agha A, Giorgi C, *et al.* Long term efficacy and toxicity of amiodarone in the treatment of refractory cardiac arrhythmias. *Cand J Cardiol* 1989; **5**: 98–104.
9. Reasor MJ, Ogle CL, Walker ER, *et al.* Amiodarone-induced phospholipidosis in rat alveolar macrophages. *Am Rev Respir Dis* 1988; **137**: 510–518.
10. Cantor JO, Osman M, Cerreta JM, *et al.* Amiodarone-induced pulmonary fibrosis in hamsters. *Exp Lung Res* 1984; **6**: 1–10.
11. Daniels JM, Brien JF, Massey TE. Pulmonary fibrosis in the hamster by amiodarone and desethylamiodarone. *Toxicol Appl Pharmacol* 1989; **100**: 350–359.
12. Wang Q, Hollinger MA, Giri SN. Attenuation of amiodarone-induced lung fibrosis and phospholipidosis in hamsters by taurine and/or niacin treatment. *J Pharmacol Exp Therap* 1992; **262**: 127–132.
13. Nakashima JM, Hyde DM, Giri SN. Inflammation, repair and fibrosis in the hamster lung following intratracheal instillation of enzyme-generated oxidants. *Exp Lung Res* 1991; **17**: 569–587.
14. Woessner JJ, Jr. The determination of hydroxyproline in tissue and protein samples containing small proportions of this imino acid. *Arch Biochem Biophys* 1961; **93**: 440–447.
15. Giri SN, Misra HP, Chandler DB, *et al.* Increases in lung prolyl hydroxylase and superoxide dismutase activities during bleomycin-induced lung fibrosis in hamsters. *Exp Mol Pathol* 1983; **39**: 317–326.
16. Ohkawa H, Ohismi N, Yagi K. Assay for lipid peroxides in animal tissues by thiobarbituric acid reaction. *Anal Biochem* 1979; **95**: 351–358.
17. Misra HP, Fridovich I. The role of superoxide anion in the autoxidation of adrenaline and a simple assay for superoxide dismutase. *J Biol Chem* 1972; **247**: 3170–3175.
18. Bligh EG, Dyer WJ. A rapid method of total lipid extraction and purification. *Can J Biochem Physiol* 1959; **37**: 911–917.
19. Ames BN, Dubin DT. The role of polyamines in the neutralization of bacteriophage deoxyribonucleic acid. *J Biol Chem* 1960; **235**: 769–775.
20. Karlinski JB, Goldstein CH. Fibrotic lung disease: a perspective. *J Lab Clin Med* 1980; **96**: 939–942.
21. Akoun GM, Cadranel JL, Blanchette G, *et al.* Bronchoalveolar lavage cell data in amiodarone-associated pneumonitis. Evaluation in 22 patients. *Chest* 1991; **99**: 1177–1182.
22. Israel-Biet D, Venet A, Caubarrere I, *et al.* Bronchoalveolar lavage in amiodarone pneumonitis. Cellular abnormalities and their relevance to pathogenesis. *Chest* 1987; **91**: 214–221.
23. Sandron D, Israel-Biet D, Venet A, *et al.* Immunoglobulin abnormalities in bronchoalveolar lavage specimens from amiodarone-treated subjects. *Chest* 1986; **89**: 617–618.
24. Kennedy TP, Gordon GB, Paky A, *et al.* Amiodarone causes acute oxidant lung injury in ventilated and perfused rabbit lungs. *J Cardiovasc Pharmacol* 1988; **12**: 23–36.
25. Li ASW, Chignell CF. Spectroscopic studies of cutaneous photosensitizing agents. IX. A spin trapping study of the photolysis of amiodarone and desethylamiodarone. *Photochem Photobiol* 1987; **45**: 191–197.
26. Lewis MS, Whatley RE, Cain P, *et al.* Hydrogen peroxide stimulates the synthesis of platelet-activating factor by endothelium and induces endothelial cell-dependent neutrophil adhesion. *J Clin Invest* 1988; **82**: 1045–2055.
27. O'Flaherty J. Neutrophil degranulation: evidence pertaining to its mediation by the combined effects of leukotriene $B_4$, platelet-activating factor and 5-HETE. *J Cell Physiol* 1985; **122**: 229–239.
28. Lin AH, Morton DR, Gorman RR. Acetylglyceryl ether phosphorylcholine stimulates leukotriene $B_4$ synthesis in human polymorphonuclear leukocytes. *J Clin Invest* 1982; **70**: 1058–1065.
29. Wardlaw AJ, Moqbel R, Cromwell O, *et al.* PAF-acether is a potent chemotactic and chemokinetic factor for human eosinophile. *J Clin Invest* 1986; **78**: 1701–1706.
30. Schaberg T, Haller H, Lode H. Evidence for a platelet-activating factor receptor on human alveolar macrophages. *Biochem Biophys Res Commun* 1991; **177**: 704–710.
31. Giri SN, Chen Z, Younker WR, *et al.* Effects of intratracheal administration of bleomycin on GSH-shuttle enzymes, catalase, lipid peroxidation and collagen content in the lungs of hamsters. *Toxicol Appl Pharmacol* 1983; **71**: 132–141.
32. Chandrakasan G, Bhatnagar RS. Stimulation of collagen synthesis in fibroblast cultures by superoxide. *Cell Mol Biol* 1992; **37**: 751–755.
33. Prockop DJ, Kivirikko KI, Tuderman L, *et al.* The biosynthesis of collagen and its disorders. *N Engl J Med* 1979; **301**: 13–23.
34. Leven CI, Bates CJ. Ascorbic acid and collagen synthesis. In: Kulonen E, Pikkarainen J, Eds. *Biology of Fibroblast*, New York: Academic Press, 1973; 397–410.
35. Kelley J, Newman RA, Evans JN. Bleomycin-induced pulmonary fibrosis in the rat. Prevention with an inhibitor of collagen synthesis. *J Lab Clin Med* 1980; **96**: 954–964.
36. Suzawa H, Kikuchi S, Arai N, *et al.* The mechanism involved in the inhibitory action of tranilast on collagen biosynthesis of keloid fibroblasts. *Jpn J Pharmacol* 1992; **60**: 91–96.
37. Caramelo C, Fernandez-Gallardo S, Santos JC, *et al.* Increased levels of platelet-activating factor in blood from patients with cirrhosis of the liver. *Eur J Clin Invest* 1987; **17**: 7–11.
38. Zhou W, Chao W, Levine BA, *et al.* Role of platelet-activating factor in hepatic responses after bile duct ligation in rats. *Am J Physiol* 1992; **263**: G587–G592.
39. Chung KF. Platelet-activating factor in inflammation and pulmonary disorders. *Clin Sci* 1992; **83**: 127–138.
40. Stenmark KR, Eyzaguirre M, Westcott JY, *et al.* Potential role of eicosanoids and PAF in the pathophysiology of bronchopulmonary dysplasia. *Am Rev Respir Dis* 1987; **136**: 770–772.
41. Valone FH. Platelet-activating factor binding to specific cell membrane receptors. In: Snyder F, Ed. *Platelet-activating factor and related lipid mediators.* New York: Plenum Press 1987; 137.
42. Peplow PV, Mikhailidis DP. Platelet-activating factor (PAF) and its relation to prostaglandins, leukotrienes and other aspects of arachidonate metabolism. *Prostag Leuko Essential Fatty Acids* 1990; **41**: 71–82.
43. Giri SN, Sanford DA, Jr., Robison TW, *et al.* Impairment in coupled beta-adrenergic receptor and adenylate cyclase system during bleomycin-induced lung fibrosis in hamsters. *Exp Lung Res* 1987; **13**: 401–416.
44. Giri SN, Nakashima JM, Curry DL. Effects of intratracheal administration of bleomycin or saline in pair-fed and control-fed hamsters on daily food intake, and plasma levels of glucose, cortisol and insulin and lung level of calmodulin, calcium, and collagen. *Exp Mol Pathol* 1985; **42**: 206–219.
45. Wang Q, Giri SN, Hyde DM. Characterization of a phospholipase-$A_2$ in hamster lung *in vitro* and *in vivo* effects of bleomycin on this enzyme. *Prostg Leuk Essential Fatty Acids* 1989; **36**: 85–92.
46. Chandler DB, Giri SN. Changes in plasma concentrations of prostaglandins and plasma angiotensin-converting enzyme during bleomycin-induced lung fibrosis in hamsters. *Am Rev Resp Dis* 1983; **128**: 71–76.
47. Giri SN, Witt TC. Effects of intratracheal administration of bleomycin on prostaglandins and thromboxane-$B_2$ and collagen levels of the lung in hamsters. *Exp Lung Res* 1985; **9**: 113–119.
48. Rodriguez-Barbero A, Boxque E, Rivas-Cabanero L, *et al.* Effect of platelet activating factor antagonist treatment on gentamicin nephrotoxicity. *Mediator Inflamm* 1992; **1**: 23–26.

ACKNOWLEDGEMENTS. This study was supported by National Heart Lung Blood Institute of NIH, Grant No. 2 RO1 HL-27354. The authors thank Dr Wang, Mary J. Schiedt, and Mary Y. Stovall for their expert technical assistance.

**Received 11 March 1993; accepted in revised form 28 April 1993**
